# Interpersonal Synchrony in the Context of Caregiver-Child Interactions: A Document Co-citation Analysis

**DOI:** 10.3389/fpsyg.2021.701824

**Published:** 2021-07-28

**Authors:** Alessandro Carollo, Mengyu Lim, Vahid Aryadoust, Gianluca Esposito

**Affiliations:** ^1^Department of Psychology and Cognitive Science, University of Trento, Rovereto, Italy; ^2^Psychology Program, School of Social Sciences, Nanyang Technological University, Singapore, Singapore; ^3^National Institute of Education, Nanyang Technological University, Singapore, Singapore; ^4^Lee Kong Chian School of Medicine, Nanyang Technological University, Singapore, Singapore

**Keywords:** attunement, biobehavioral synchrony, CiteSpace, document co-citation analysis, neural synchronization, systematic review, synchrony, social interactions

## Abstract

Social interactions accompany individuals throughout their whole lives. When examining the underlying mechanisms of social processes, dynamics of synchrony, coordination or attunement emerge between individuals at multiple levels. To identify the impactful publications that studied such mechanisms and establishing the trends that dynamically originated the available literature, the current study adopted a scientometric approach. A sample of 543 documents dated from 1971 to 2021 was derived from Scopus. Subsequently, a document co-citation analysis was conducted on 29,183 cited references to examine the patterns of co-citation among the documents. The resulting network consisted of 1,759 documents connected to each other by 5,011 links. Within the network, five major clusters were identified. The analysis of the content of the three major clusters—namely, “Behavioral synchrony,” “Towards bio-behavioral synchrony,” and “Neural attunement”—suggests an interest in studying attunement in social interactions at multiple levels of analysis, from behavioral to neural, by passing through the level of physiological coordination. Furthermore, although initial studies on synchrony focused mostly on parent-child interactions, new hyperscanning paradigms are allowing researchers to explore the role of biobehavioral synchrony in all social processes in a real-time and ecological fashion. Future potential pathways of research were also discussed.

## 1. Introduction

Social interactions and relationships constitute one of the major forces that continuously and dynamically shape human development throughout the lifespan. From classical observational studies, recent advances in the techniques employed in social neuroscience enabled researchers to take the study of human sociability to a new level of understanding that strongly highlights interpersonal multi-level synchronization processes. These recent developments on the study of social interactions and, specifically, the role played by synchrony, represent the major interest of the current paper.

Social interactions are crucial for humans since the first days of life. Not only do newborns largely depend on significant others in order to satisfy their fundamental needs, but the quality of early relational exchanges influence the infants' subsequent emotional, cognitive and social development (Belsky and Fearon, 2002; Malekpour, 2007; Groh et al., 2017; Bell, 2020; Cataldo et al., 2021). For this reason, studying the quality of early interactions has become the key to understanding typical and atypical trajectories that can emerge in the individual's development (Harrist and Waugh, 2002; Bertamini et al., 2021). Among others, synchrony proved useful in assessing relational quality (Leclère et al., 2014). When applied to the behavioral analysis of social interactions, synchrony, which is central in the intersubjectivity theory, refers to the temporal relationship between events so that dyadic behaviors become coordinated (Beebe and Gerstman, 1980; Tronick, 1989; Feldman, 2007; Zampella et al., 2020). In this framework, initial observational studies on behavioral coordination suggested that early interactions between mother and infant depend on both members tuning to each other's signals (Belsky, 2009). By doing so, each individual displays a set of adaptive and reciprocal behaviors that promotes a mutually rewarding interaction (Reyna and Pickler, 2009). To a broader extent, a higher degree of shared emotions, mutual engagements and turn-taking is characteristic of all dyadic interactions where partners are sensitively attuned to each other (Ambrose and Menna, 2013; Azhari et al., 2019). This attunement seems associated with increased relational quality, cooperation, empathy, social cognition, and smoother conversations (Hove and Risen, 2009; Zampella et al., 2020).

In human social contact, coordinated behaviors are associated with coordinated, or attuned, physiological responses (Feldman, 2012a). Such attunement starts in utero and is believed to be the mechanism that allows the parent, in the early stages of life, to exert an exogenous control on the child's physiology in order to maintain an internal state of allostasis (Bauman, 2000; Rao et al., 2013; Nguyen et al., 2020b). Several systems, such as body temperature (Levin, 2006), immune functions (Arrieta et al., 2014), and heart rate (Feldman et al., 2011) emerged to be involved in the physiological attunement between mother and child. Recent advances in social neuroscience techniques (i.e., hyperscanning studies) has broadened this group of physiological systems to include neural signals (Koike et al., 2015; Reindl et al., 2018). From existing literature, it emerged that biobehavioral synchrony (or attunement)—the temporal coordination of behavioral and physiological events (Atzil et al., 2012; Azhari et al., 2020c)—is a pervasive mechanism underlying human sociability. The experience of synchrony in early phases of life seems to enable the cross-generation transmission of attachment patterns (Feldman, 2007; Feldman et al., 2010).

### 1.1. The Current Study

Research in biobehavioral synchrony is gaining momentum in recent years and a considerable set of knowledge is emerging with promising results in the field of social neuroscience. To provide insight into the research that have shaped the comprehension of human social interactions from the perspective of synchrony, the current study adopted a scientometric approach. Of particular interest were two aspects: (i) identifying impactful contributions in the field and (ii) examining main research trends that shaped the available literature. This paper serves as a systematic organization of prevailing literature and as a milestone summary and evaluation of research directions in this field thus far. It is hoped that the paper may lend a macro perspective to researchers in this field on our current understanding of interpersonal synchrony, chart out potential future research directions and identify the still unanswered questions.

## 2. Materials and Methods

**Figure 1:**
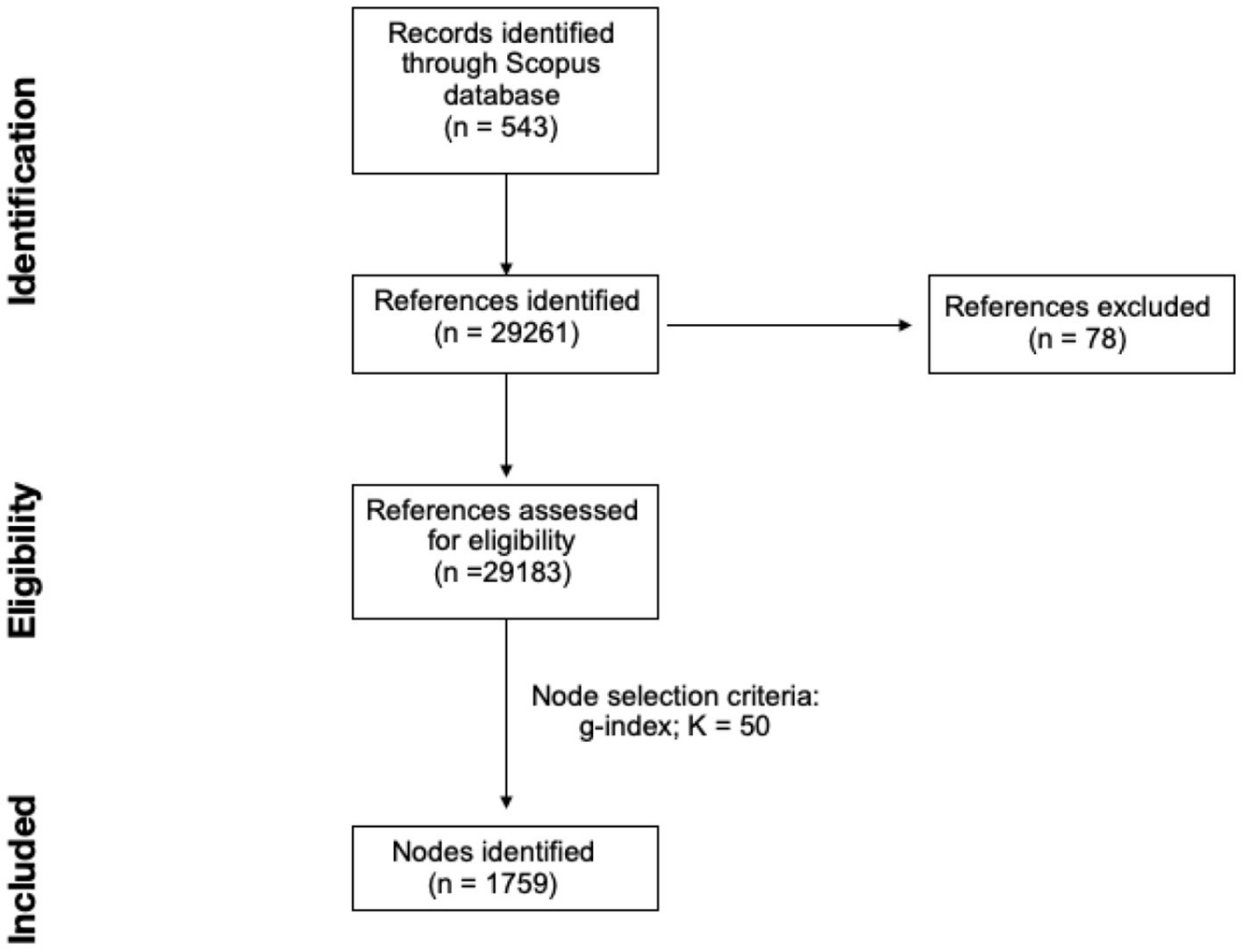
Study flow diagram.

As done in previous scientometric reviews (Carollo et al., 2021b; Rawat and Sood, 2021), the sample of publications used in this study was obtained from Scopus. In particular, the bibliographic search used the following string of keywords: “TITLE-ABS-KEY ((“attunement” OR tun* OR symmetr* OR syncron* OR “turn-taking” OR “brain-to-brain” OR “hyperscann*”) AND (“social interaction” OR “social behavio*” OR sociality OR “mother-child”) AND (child* OR infan* OR caregiv* OR parent* OR peer*)) AND (LIMIT-TO (LANGUAGE, “English”)).” The single keywords were selected to cover and collect the larger amount of literature regarding mechanisms of attunement and synchrony in social contexts, with a focus on the early social interactions. For this reason, in the string we included: (i) a methodological component with synonyms of synchrony commonly used in the scientific literature; (ii) a contextual component that directed the research to the social interactions world; and (iii) a population component that focus the attention on children and parents, and, more generally, peers to explore the social world to a larger extent. The sample consisted 543 qualified documents published between 01 January 1971 and 22 March 2021. This range of time depended entirely on Scopus' document availability, for no temporal criteria was applied to the bibliographic search conducted on 22 March 2021. CiteSpace software (version 5.7.R2) was used for scientometric analysis and, when importing the dataset of publications, 29,183 references over a total of 29,261 (99.73%) were considered valid. Successful conversion when importing data on CiteSpace has a possible data loss of ~1.0–5.0%, which typically depends on data irregularity. Thus, the small percentage of data loss that occurred in the study (0.27%) can be considered as negligible (see Figure 1) (Gaggero et al., 2020).

To examine the main trends in the literature regarding interpersonal synchrony in social interactions, a Document Co-Citation Analysis (DCA) was computed. This is a type of analysis based on the frequency with which two or more documents are cited together, or co-cited (Small, 1980). DCA is rooted in the hypothesis that frequent co-citations among documents reflect common research trends and intellectual domains within the available literature (Chen et al., 2010). Based on these principles, the network that results from DCA is composed of documents that are frequently cited together as well as the documents that cite them (which, in this case, are all from Scopus).

To create a balanced network of documents, several DCAs were computed and compared using three different node selection criteria, namely g-index, TOP N and TOP N%, as in Carollo et al. (2021a). G-index was introduced as an improvement of the h-index, for it better takes into account the citation scores of an author's top publications (Egghe, 2006; Alonso et al., 2009). Specifically, the g-index represents the “largest number that equals the average number of citations of the most highly cited g publications” (Chen, 2016). Conversely, TOP N and TOP N% are criteria that select all the N or N% most cited documents within a time slice (which in this study was always kept at the value of 1 year) as network nodes (Chen, 2014). Not only were node selection criteria varied at this stage, but so were their scale factor values. In particular, g-index with k set at 75, 50, 25, 15, and 10, TOP N with N at 50, 15, and 10, and TOP N% with N at 50 and 10 were tested. The overall effects on the network's structural metrics and the number of nodes and clusters identified were weighted for the final decision on the node selection criteria and the scale factor's value to use in the DCA. Eventually, the best criteria emerged as the g-index with k at 50 and, therefore, it was used to generate the network of documents.

In CiteSpace, results are described with two parameters: structural and temporal metrics. The former category, structural metrics, encompasses modularity Q, silhouette score and betweenness centrality indexes. Modularity Q, with its values ranging from 0 to 1, indicates the degree to which the network can be decomposed into single groups of nodes, also called modules or clusters (Newman, 2006). High values of modularity Q imply a well-structured network (Chen et al., 2010). Next, silhouette score measures the inner consistency (i.e., cohesion and separation) of the modules into which the network is divided (Rousseeuw, 1987). Values of silhouette range from −1 to 1, with larger values representing a cluster's high separation from other modules as well as internal consistency (Aryadoust and Ang, 2019). The last structural metric, namely betweenness centrality, represents the degree to which a node functions as a bridge to connect an arbitrary pair of nodes in the network (Freeman, 1977; Chen, 2014). Its values range from 0 to 1, which is typically obtained by groundbreaking and revolutionary works in the scientific landscape (Aryadoust et al., 2019). The other group of metrics—temporal metrics—includes citation burstness and sigma. Citation burstness, which is calculated through Kleinberg's algorithm (Kleinberg, 2003), indicates an abrupt increase in the number of citations that a document has received within a given period of time (Chen, 2017). Lastly, sigma metric, computed with the equation (centrality+1)^*burstness*^, gives information on a document's novelty and its influence on the overall network (Chen et al., 2009). Influential publications have higher citation burstness and sigma. In this study, structural metrics, in particular modularity Q and silhouette score, were used to examine the overall configuration of the network and clusters. Additionally, properties of single nodes were examined using both structural, specifically betweenness centrality, and temporal metrics.

## 3. Results

DCA resulted in a network composed of 1,759 nodes (cited and citing documents) and 5,011 links. Thus, on average, each node showed 2.85 connections with other nodes in the network. A modularity Q of 0.971 indicates that the network was highly divisible into clusters, and an average silhouette score of 0.9879 indicates that each cluster was highly consistent. To testify to the novelty of this field of research, only seven documents showed a citation burst in the network, which gives a measure of a paper's relevance in the scientific panorama (see details in Table 1). Specifically, the DSM-5 (American Psychiatric Association, 2013) was linked with a citation burst of 11.31 lasting from 2015 to 2021, indicating strongest citation burst metrics for the longest period of the network. While DSM-5 belonged to cluster #15, three of the other six documents was included in cluster #0 and the other three in cluster #1. Specifically, the documents showing a citation burst in cluster #0 were: Feldman (2012a) (strength of burst = 3.67; burst duration = 3), Atkinson et al. (2013) (strength of burst = 3.37; burst duration = 4), and Feldman et al. (2011) (strength of burst = 2.83; burst duration = 2). Conversely, the publications with a citation burst in cluster #1 were: Reindl et al. (2018) (strength of burst = 2.97; burst duration = 2), Jiang et al. (2012) (strength of burst = 2.97; burst duration = 2), and Babiloni and Astolfi (2014) (strength of burst = 2.9; burst duration = 4).

**Table 1:** Identifying characteristics of the 7 publications with high citation burstness metrics generated in the DCA.

**References**	**Strength of burstness**	**Year**	**Beginning of burstness**	**End of burstness**	**Burst duration**
DSM-5 (American Psychiatric Association, 2013)	11.31	2013	2015	2021	6
Feldman (2012a)	3.67	2012	2016	2019	3
Atkinson et al. (2013)	3.37	2013	2015	2019	4
Reindl et al. (2018)	2.97	2018	2019	2021	2
Jiang et al. (2012)	2.97	2012	2019	2021	2
Babiloni and Astolfi (2014)	2.9	2014	2017	2021	4
Feldman et al. (2011)	2.83	2011	2015	2017	2

**Figure 2:**
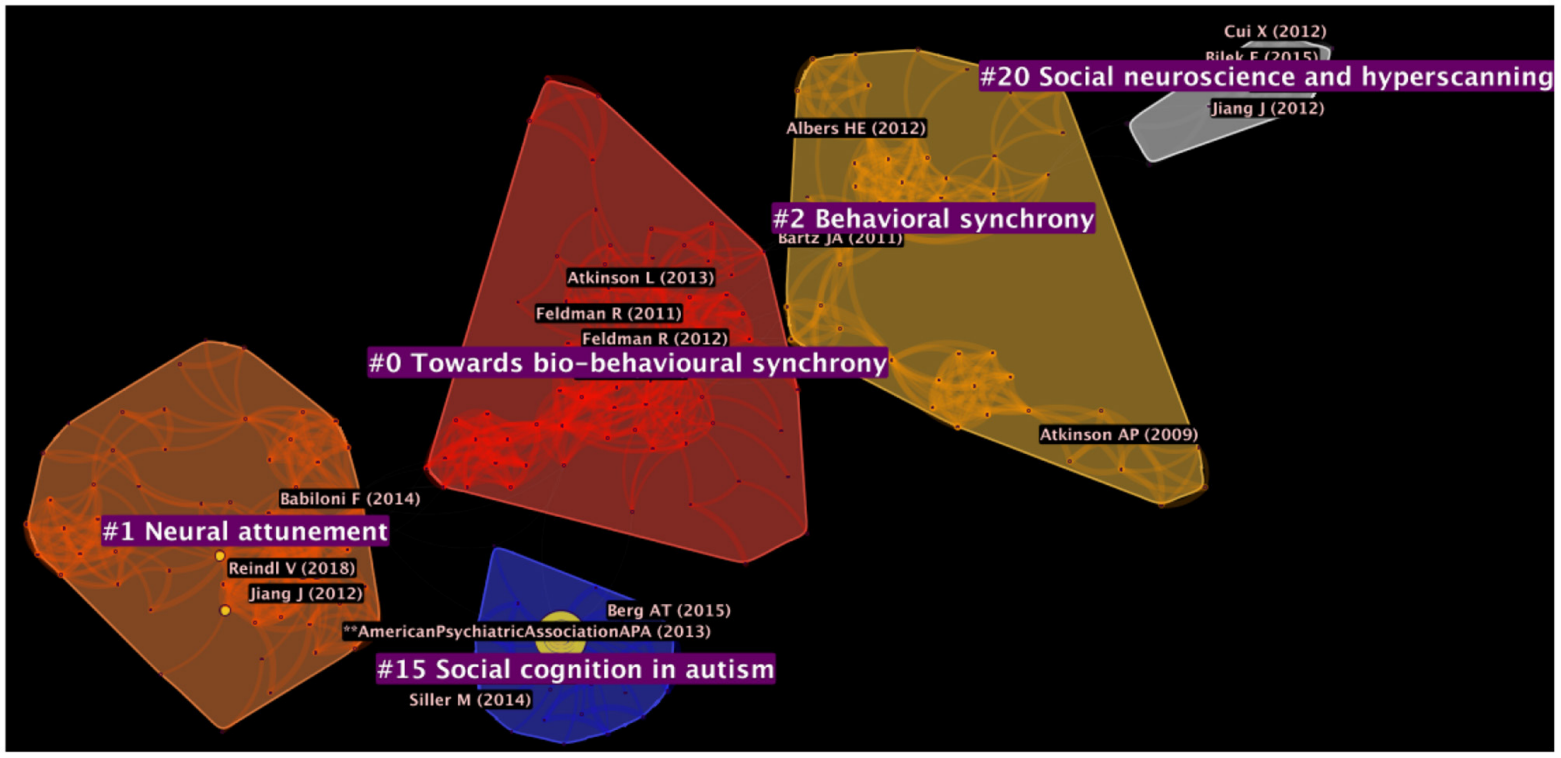
Network of publications generated through the DCA.

Five major clusters were identified within the network of documents (see details in Figure 2 and Table 2). These clusters had an average year of document publication around the 2010s. Chronologically, documents included in cluster #2 were, on average, published in 2008. This cluster was the only one with an average year of publication in the 2000s. Other clusters emerged more recently and included cluster #0 and cluster #20 (both with average year of publication = 2012), cluster #15 (average year of publication = 2013) and, finally, cluster #1 (average year of publication = 2016), which was the most recent. With regards to the size of the clusters, cluster #0 was the largest within the network, consisting of 73 documents with a silhouette score of 0.988. Cluster #0 was followed by cluster #1 (size = 62; silhouette = 0.995) and cluster #2 (size = 53; silhouette = 0.985). Generally, all clusters were recent and highly internally homogeneous. The clusters are automatically labeled by the log-likelihood ratio (LLR) algorithm of CiteSpace. Previous research shows that LLR provides the most accurate labeling of clusters among other available methods in CiteSpace, although these can be imprecise. Then, we read the content of the major publications in each cluster to verify the LLR labels, discussed below.

**Table 2:** Metrics of the 5 clusters identified with the Document Co-citation Analysis (DCA).

**Cluster ID**	**Size**	**Silhouette**	**Mean year**	**LLR label**	**Proposed label**
0	73	0.988	2012	Affiliative neuroscience approach	Towards bio-behavioral synchrony
1	62	0.995	2016	Studying parent-child interaction	Neural attunement
2	53	0.985	2008	Oxytocin pathway	Behavioral synchrony
15	24	0.98	2013	Autism spectrum disorder	Social cognition in autism
20	16	0.985	2012	Oxytocin pathway	Social neuroscience and hyperscanning

## 4. Discussion

This study set out to investigate mechanisms of biobehavioral synchrony in early social interactions through a scientometric approach. To do so, we employed DCA to identify research clusters, scientific domains, that, in a dynamic fashion, contributed to give shape to the available literature of this field. The scientific contribution of the three major clusters is discussed below following the chronological order of the average year in which the related documents were published. Each cluster was named by using the CiteSpace's LLR naming option.

### 4.1. Cluster #2: “Behavioral Synchrony”

Table 3 reports the three citing documents in cluster #2 with their global citing score (GCS; total number of citations a paper received in Scopus), local citing score (LCS; total citations of the citing paper in the dataset), and coverage (number of references in the cluster that were cited by the citing documents). Collectively, these three publications are considered key contributors for the formation of the cluster and were therefore examined closely.

**Table 3:** Citing documents in cluster #2 identified using the DCA.

**Cluster**	**Citing document**	**GCS**	**LCS**	**Coverage**
2	Carter (2014)	291	1	11
2	Flinn (2010)	3	1	10
2	Leclère et al. (2014)	145	1	8

*Values of global citing score (GCS), local citing score (LCS) and coverage are reported for the citing documents. GCS stays for the total number of citations a paper received in Scopus. LCS indicates the number of citations a paper received in the dataset of the current study. Coverage refers to the number of documents in the cluster that were cited by the paper*.

With their systematic review, Leclère et al. (2014) focused on the concept of synchrony, here intended as co-regulation of behaviors between individuals, and the way in which it has been operationalized often times by using different terms, such as mutuality, reciprocity, rhythmicity, harmonious interaction, turn-taking and shared affect and scientifically examined over the years. Specifically, the authors defined behavioral synchrony as “a dynamic and reciprocal adaptation of the temporal structure of behaviors and shared affect between interactive partners,” typically represented by the mother-child dyad. Synchrony also refers to the process in which hormonal, physiological, and behavioral cues are exchanged between the dyad (Feldman, 2012b). In the majority of the reviewed studies, mother-child interactions were video-recorded. Three main methods for the measurement of behavioral synchrony were identified: first, global interaction scales with dyadic items, as in Abraham et al. (2014); second, specific synchrony scales; and finally, micro-coded time-series analyses, which are based on a statistical approach (e.g., computing the frequency of specific mother and/or infant behaviors).

Overall, the analyzed literature suggests that beneficial outcomes are associated with higher mother-child behavioral coordination, such as the sense of familiarity and healthy mother and child development. In fact, the mother's readiness to respond to the child's needs contributes to modifying infant behavioral and physiological responses (Zhang and Meaney, 2010). For instance, Ambrose and Menna (2013) observed that interpersonal behavioral synchrony in mother-child free play sessions is negatively associated with child physical aggression. Furthermore, lower coordination and responsiveness in dyadic processes can be due to maternal depression (Barrett and Fleming, 2011), as shown by Katherine Weinberg et al. (2006) using the Face-to-Face Still-Face paradigm with 3-month old infants. The same effect is less clear when examining the impact of childbirth-related post traumatic stress disorder (PTSD) (Alcorn et al., 2010) on parent-child bond, with studies obtaining contrasting results. In fact, while Ayers et al. (2006) reported an association between PTSD symptoms and parent-child bond, Ayers et al. (2007) did not replicate the results, asking for further research to tackle some methodological considerations, by using, for instance, a more detailed measure of the bond between parent and baby.

At a molecular level, the article by Leclère et al. proposes that oxytocin is able to enhance physiological and behavioral readiness for social engagement in parent-infant interactions (Leclère et al., 2014), through biological mechanisms that are not fully understood yet. Similarly, in the review by Carter (2014), the authors propose that oxytocin, by creating a sense of safety, facilitates social sensitivity and attunement to rearing practices and, more generally, human sociability. Oxytocin involvement in parental attachment was also confirmed by other cited studies in the cluster (Bartels and Zeki, 2004; Feldman, 2012b). Due to the interest in this nonapeptide, many studies exploring its property were also cited and included in the network (Bales et al., 2004, 2007, 2013, 2014; Apicella et al., 2010; Bick and Dozier, 2010; Bartz et al., 2011; Meyer-Lindenberg et al., 2011; Weisman et al., 2013; Arrowsmith and Wray, 2014).

Finally, the cluster also included a hyperscanning study by Dumas et al. (2010), where brain synchrony during ongoing social interactions was assessed with a dual- electroencephalography (EEG) setup.

### 4.2. Cluster #0: “Towards Bio-Behavioral Synchrony”

The seven citing documents within cluster #0 are reported in Table 4, with details on GCS, LCS and coverage. For their relevance in shaping the cluster, these publications, together with other relevant cited nodes, were examined closely.

**Table 4:** Citing Citing documents in cluster #0 identified using the DCA.

**Cluster**	**Citing document**	**GCS**	**LCS**	**Coverage**
0	Feldman (2020)	14	1	13
0	Abraham et al. (2016)	28	1	10
0	Feldman (2015b)	104	1	8
0	Saxbe et al. (2015)	6	1	7
0	Hibel et al. (2015)	40	1	6
0	Atkinson et al. (2016)	20	1	6
0	Levy et al. (2016)	37	1	5

*Values of global citing score (GCS), local citing score (LCS) and coverage are reported for the citing documents. GCS stays for the total number of citations a paper received in Scopus. LCS indicates the number of citations a paper received in the dataset of the current study. Coverage refers to the number of documents in the cluster that were cited by the paper*.

Unsurprisingly, similar to cluster #2, behavioral coordination (or synchrony) in parent-infant interactions seems to predict infant attachment style (Beebe et al., 2010). At the neural level, exposure to functional relational exchanges, when compared to more pathological ones, elicits particular patterns of brain activation in healthy postpartum mothers, suggesting that synchronous and non-synchronous interactions are processed differently (Atzil et al., 2014). Specifically, when watching infant-related vignettes, behaviorally synchronous mothers have higher activation in the left nucleus accumbens, which in turn is functionally connected to regions linked to emotion moderation, theory-of-mind and empathy. Conversely, intrusive and less behaviorally synchronous mothers show greater activity in the right amygdala (Atzil et al., 2011). From the literature, it emerged that the parent's degree of functional connectivity in the embodied simulation network enables parents to attune to the infant state and emotions (Feldman, 2017), predicting lower levels of cortisol in the infant (Abraham et al., 2018).

This is possible because parent-child synchrony not only takes place at the behavioral level, but also in a physiological fashion (Feldman et al., 2011). The study by Hibel et al. (2015) examined adrenocortical synchrony between mothers and their children. Adrenocortical synchrony refers to the coordination between the child's adrenocortical activity and maternal behavior and cortisol levels (see also Albers et al., 2008; Ruttle et al., 2011). It is to note that physiological synchrony is not a continuous steady state and we are far from reaching a full understanding of the mechanisms that control and regulate it as the dyad adjust to the world's demands. Nevertheless, some studies suggest that this physiological co-regulation could be the physiological result of the dyad's shared experiences (Feldman, 2007; Papp et al., 2009). What Hibel et al. observed is that adrenocortical synchrony is moderated by both members of the dyad, for it depends on both maternal sensitivity and the child's emotional reactivity (as documented in Middlemiss et al., 2012; Atkinson et al., 2013). For this reason, maternal depression, which can undermine maternal sensitivity, is associated with less optimal child stress response and developmental outcomes (Feldman et al., 2009; Barker et al., 2011; Laurent et al., 2011; Apter-Levi et al., 2016; Granat et al., 2017; Pratt et al., 2019). A related study by Azhari et al. (2020b) found that maternal anxious attachment is slightly, albeit non-significantly, correlated with decreased brain-to-brain synchrony between mothers and their infants using functional near-infrared spectroscopy (fNIRS). Similarly, only infants with disorganized attachment show higher cortisol levels in stressful situations compared with playful situations (Bernard and Dozier, 2010).

Adrenocortical synchrony is not limited to early childhood, for it has been observed in adolescents (Saxbe et al., 2015). This synchrony is also reflected at the neural level in regions involved in social cognition (i.e., precuneus, posterior cingulate and retrosplenial cortex), in response to parent-related stimuli. Nevertheless, from the review by Atkinson et al. (2016), methods of assessment of HPA axis functions in the context of parent-child adrenocortical synchrony emerge to be quite overly focused on individual reactivity to single and selected laboratory tasks, providing scarce evidence in different settings, challenges and experimental designs (see Alexander et al., 2011; Allwood et al., 2011; Ali and Pruessner, 2012; Andrews et al., 2013; Bernard et al., 2017).

A combination of multiple levels of synchrony led to the concept of biobehavioral synchrony, referring to the mechanisms through which the early environment exerts its influences by coordinating biological and social processes during social contact (Feldman, 2012a; Abraham et al., 2016). Within this conceptual framework, Feldman (2015b) explored how biobehavioral synchrony interacts with the child's oxytocin system. In Feldman (2020), this is suggested to be crucial for resilience later in life. The close relation between the HPA axis and oxytocin system may allow the latter to function as a buffer to mitigate consequences of environmental stress on the developing brain (Neumann, 2008). Studies examining the properties of oxytocin were included in the clusters (Numan, 2012; Anacker and Beery, 2013; Apter-Levy et al., 2013; Feldman, 2015a; Feldman et al., 2016).

Not only is biobehavioral synchrony thought to be relevant to the parent-child dyad, but also to the mother-father dyad in human bi-parental rearing. Within this context, Atzil et al. (2012) studied brain synchrony (or coordination of brain activity) in coupled mother and father dyads. The authors employed functional magnetic resonance imaging (fMRI) to study brain activation of each member of the dyad when exposed to infant-related videos. Results showed a coordinated pattern of activity within the social-cognitive networks, the basis of parental sensitivity, among the members of the dyad. In a related study, Azhari et al. (2020c) studied spousal brain-to-brain synchrony using fNIRS when exposed to infant-related vocalizations. It was found that spouses who were in physical presence with each other displayed unique patterns of synchronization that were absent when they were physically apart or when random non-related mother-father dyads were paired. Although these results provide initial evidence suggesting a brain-to-brain synchronization between individual in an attachment relationships, the role that this synchrony plays in bonding formation and, ultimately, practices of co-parenting is still to be clarified (Atzil et al., 2012).

More generally, at a pure methodological level, the cluster included several articles in which methods to assess brain synchrony were explored. For instance, these methodologies included simultaneous EEG recordings (Babiloni et al., 2012), magnetoencephalography (MEG) (Baess et al., 2012) and fMRI dual scanning during real-time social interactions (Bilek et al., 2015). Although they allow a simultaneous measurements of multiple brains, these instruments did not consent the assessment of neural synchrony in a naturalistic approach. In fact, with these instruments, participants are still examined in a laboratory setting and with almost no movement allowed.

### 4.3. Cluster #1: “Neural Attunement”

The four citing documents within cluster #1 are reported in Table 5, which provide details on GCS, LCS and coverage. These and other references that strongly contributed to form the cluster were examined closely.

**Table 5:** Citing documents in cluster #1 identified using the DCA.

**Cluster**	**Citing document**	**GCS**	**LCS**	**Coverage**
1	Nguyen et al. (2020a)	3	1	19
1	Nguyen et al. (2020b)	20	1	14
1	Nguyen et al. (2020c)	0	1	10
1	Santamaria et al. (2020)	8	1	8

*Values of global citing score (GCS), local citing score (LCS) and coverage are reported for the citing documents. GCS stays for the total number of citations a paper received in Scopus. LCS indicates the number of citations a paper received in the dataset of the current study. Coverage refers to the number of documents in the cluster that were cited by the paper*.

A node in the cluster is represented by Bizzego et al. (2021), where the authors showed that physiological synchrony is influenced by the type of relationship between the dyad. Considering the role that biobehavioral synchrony plays in promoting the child's social capacities (Atzil and Gendron, 2017), Baker et al. (2015) observed that electrodermal synchrony between parent and child is moderated by symptoms of autism. In other words, electrodermal synchrony is higher for dyads with children who have lower autism symptom levels.

An innovative shift in perspective emerged in neuroscience around the 2010s, promoted by several studies in the cluster. This approach, called “second-person neuroscience approach,” is characterized by the attempt to study human sociability by using experimental paradigms designed to involve participants in structured or ecologically valid, real-time reciprocal social interactions (Schilbach et al., 2013; Bolis and Schilbach, 2018; Hoehl and Markova, 2018; Redcay and Schilbach, 2019). Although the majority of classical cognitive studies focus on the processes happening within the single individual, evidence suggests that neural activities of two brains can be coordinated due to signals transmitted through the environment (e.g., face-to-face social interactions) (Hasson et al., 2012; Jiang et al., 2012; Liu et al., 2016; Hoehl et al., 2021). This phenomenon—namely, neural synchrony—is believed to be the mechanism supporting effective communication and behavioral coordination among individuals (Nguyen et al., 2020b).

In this theoretical context, we can frame the emergence of hyperscanning studies, discussed in the review by Babiloni and Astolfi (2014). This approach, based on the simultaneous recording of different individuals' brain activities, allows the measurement of inter-brain correlations (as in Nozawa et al., 2016; Balconi and Vanutelli, 2018; Bevilacqua et al., 2019). Thus, hyperscanning fits well in the study of interpersonal dynamics (Nguyen et al., 2020a). By using the hyperscanning approach, some promising results have emerged. For instance, it seems that individuals show greater brain-to-brain neural synchrony when they take part in cooperative activities (Cui et al., 2012; Liu et al., 2016; Fishburn et al., 2018). In joint play, theta neural oscillations in mothers' brains predict their 12-month old infants' attention when adopting a dual-EEG recording (Wass et al., 2018). Distinct patterns of brain synchronization emerge depending on the gender of the people composing the dyads, even though some studies report higher neural syncrony for same-sex dyads than mixed-sex ones, while other studies observed the opposite pattern (Cheng et al., 2015; Baker et al., 2016). The understanding of such gender-specificity and its influence on performances demands for future studies. Not only does neural synchronization vary depending on the individuals' genders, but, as observed by Bizzego *et al*. for physiological synchrony, it changes depending on the type of relationship existing between the individuals. For instance, Pan et al. (2017) reported that, in cooperative contexts, female-male lover dyads display higher inter-brain synchronization in right superior frontal cortex, when compared to other types of dyads, such as female-male friends or strangers.

Brain-to-brain synchronization assessed with dual-functional near-infrared spectroscopy (fNIRS) paradigms can be observed in parent-child dyads, and not in stranger-child ones (Reindl et al., 2018). In particular, Nguyen et al. (2020b) found neural synchronization between mothers and children in the bilateral prefrontal cortex and temporo-parietal regions during cooperation tasks. Moreover, genders of the individuals within the dyad seem to modulate the patterns of synchronization. Specifically, mother-son dyads, when compared to mother-daughter dyads, display lower neural synchronization in the independent task and greater synchronization in the cooperative one Miller et al. (2019). Apart from these differences, in general, neural synchrony in cooperative situations appears to be positively associated with behavioral reciprocity and predicts problem-solving success (Nguyen et al., 2020b; Hoehl et al., 2021). For this reason, Nguyen et al. (2020b) suggested considering neural synchrony as a biomarker for the quality of mother-child social interactions.

In the same year, Nguyen et al. (2020c) tried to extend such results to father-child social interactions. The authors observed neural synchronization between fathers and children's brains in bilateral dorsolateral prefrontal cortex and the left temporo–parietal junction during cooperation. Neural synchrony between brains was positively associated with the father's attitude toward his parental role, which, in turn, is linked to reduced child psychopathology (Barker et al., 2017). Similarly, a preprint produced by Azhari et al. (2020a) found unique father-child synchronization in the medial left region of the brain as compared to non-related father-child dyads during co-viewing of emotionally arousing stimuli. Evidence suggests that the neural synchronization in parent-child dyads depends on the emotional quality and tone adopted in the interactions (Santamaria et al., 2020), and it is undermined by parental stress (Azhari et al., 2019). It is hypothesized that synchronization between parent and child neural activities might reflect the emotional connection within the dyad and seems to be associated with the child's development of adaptive strategies in regulating emotions (Reindl et al., 2018; Quiñones-Camacho et al., 2020).

To conclude, this alignment of neural activity between adult and child seems to depend on rhythmical social signals happening during communicative exchange that stimulates reciprocity between children and their caregivers (Leong et al., 2017; Markova et al., 2019). Nevertheless, further researches are required to understand the several fine ways in which synchrony at all level of analysis emerges and is dynamically modulated.

## 5. Conclusion

The current article explored the main scientific trends and individual papers' contributions to the field of interpersonal synchrony in social interactions, particularly within the parent-child context. A scientometric approach was adopted that led to the identification of three major clusters that represent scientific domains within the available literature. These three main clusters were all located around the years 2010s and 2020s, and were, in chronological order, “Behavioral synchrony,” “Towards bio-behavioral synchrony,” and “Neural attunement.” Some main trends of research emerged when examining the documents. In particular, the study of interpersonal synchrony in social interactions was strongly directed to explore the relation between parent (in most cases the mother) and their child. The analysis of synchrony evolved from purely behavioral level (cluster #2) to the neural analysis, largely based on hyperscanning paradigms (cluster #1). This was not an abrupt shift, for the transition from behavioral to neural levels passed through the stage of physiological attunement, where special attention was given to the activity of the HPA axis (cluster #0).

When interpreting the results of this scientometric review, it is worth noting that there are some methodological limitations to consider. First, the review depends on keywords that were used at the initial stage to drive the bibliographic research. Thus, some key terms indexing documents that represent a significant contribution in the literature concerning interpersonal synchrony in social interactions may have not been captured and excluded unintentionally from the review. Nevertheless, one document is usually indexed by several keywords, and thus it is very likely that a large part of the relevant documents in the literature were included in this review. A second limitation might emerge when considering the platform that was used to collect the documents, Scopus. Future works might extend the present review by using data derived from different platforms, such as Web of Science. Finally, at a theoretical level, the final limitation is that the scientometric approach of DCA depends on the quantitative patterns of citation (and co-citations) among documents. This leads to two further, more specific considerations. First, in a scientometric analysis, citations are treated all at the same way without any insight of their effective nature or the reasons behind each citation (e.g., whether a document is cited because it shows replicated or, conversely, controversial results). The second consideration is that the impact of recent influential publications might have been underestimated or even ignored because they were not yet massively cited by the documents in the data pool. When using this methodological approach, there is an inevitable bias toward past documents for their higher number of citations, due to their longer “lifetime” since their publication date.

These limitations notwithstanding, the results testify that research concerning interpersonal synchrony in social interactions is evolving rapidly. Novel hyperscanning paradigms that grew from the second-person neuroscience approach are starting to show their potential on understanding how all kinds of social interactions dynamically unfold in ecological contexts. As seen in cluster #1, this trend of research is allowing scholars to document, even at the inter-personal level, different types of social interactions processed in different ways. The content discussed in the current review may help future works define new pathways of research to better understand how synchrony in social interactions works. For instance, some studies are starting to clarify the mechanisms and the conditions required in order to observe synchrony, such as having face-to-face interaction. Not only is it important to investigate the environmental properties that triggers synchrony, but also the risk factors that may disrupt it, such as postpartum depression in mother-infant interactions. This is especially relevant when considering that synchrony in parent-child interactions seems to play a role on the child's social and emotional development.

To conclude, the paper was written in an attempt to systematically organize and present the main themes of research related to interpersonal synchrony, particularly within the parent-child context. From the results, we have uncovered that the main directions of synchrony research were driven by technological advancements: transitioning from behavioral, to physiological, and finally to neurological measurements of interpersonal synchrony. Research in this field has also developed to become more ecologically valid, with more and more studies focusing on naturalistic interactions and the conditions through which interpersonal synchrony can be achieved. Moving forward, it is expected that more influential future directions in this field may be taken as the tools of measuring synchrony become more sophisticated and allowing for data triangulation and the consideration of synchrony from different approaches, and findings applied to other interpersonal contexts and a wide spectrum of social situations. Research on interpersonal synchrony remains a relatively young and burgeoning area of scientific inquiry, with much potential in the social sciences.

## Data Availability Statement

The raw data supporting the conclusions of this article will be made available by the authors, without undue reservation.

## Author Contributions

AC and GE: conceptualization. AC: methodology, formal analysis, investigation, data curation, visualization, and writing—original draft preparation. AC, ML, VA, and GE: writing—review and editing. GE: supervision and funding acquisition. All authors have read and agreed to the published version of the manuscript.

## Conflict of Interest

The authors declare that the research was conducted in the absence of any commercial or financial relationships that could be construed as a potential conflict of interest.

## Publisher's Note

All claims expressed in this article are solely those of the authors and do not necessarily represent those of their affiliated organizations, or those of the publisher, the editors and the reviewers. Any product that may be evaluated in this article, or claim that may be made by its manufacturer, is not guaranteed or endorsed by the publisher.
